# Partially Etched Piezoelectric Film Filled with SiO_2_ Structure Applied to A1 Mode Resonators for Transverse Modes Suppression

**DOI:** 10.3390/mi14091745

**Published:** 2023-09-07

**Authors:** Zhenyi Yu, Yu Guo, Sulei Fu, Baichuan Li, Peisen Liu, Shuai Zhang, Zongqin Sun

**Affiliations:** 1 School of Internet of Things Engineering, Jiangnan University, Wuxi 214122, China; yzy602841972@163.com (Z.Y.); 6211922014@stu.jiangnan.edu.cn (S.Z.); 19826382131@163.com (Z.S.); 2 Key Laboratory of Advanced Materials (MOE), School of Materials Science and Engineering, Tsinghua University, Beijing 100084, China; suleifu@163.com (S.F.); lps21@mails.tsinghua.edu.cn (P.L.); 3 College of Information, Mechanical and Electrical Engineering, Shanghai Normal University, Shanghai 200234, China; 1000494740@smail.shnu.edu.cn

**Keywords:** A1 mode resonator, transverse modes suppression, partially etched piezoelectric film, comparison, finite element method

## Abstract

With the arrival of the Fifth Generation (5G) communication era, there has been an urgent demand for acoustic filters with a high frequency and ultrawide bandwidth used in radio-frequency (RF) front-ends filtering and signal processing. First-order antisymmetric (A1) lamb mode resonators based on LiNbO_3_ film have attracted wide attention due to their scalable, high operating frequency and large electromechanical coupling coefficients (*K*^2^), making them promising candidates for sub-6 GHz wideband filters. However, A1 mode resonators suffer from the occurrence of transverse modes, which should be addressed to make these devices suitable for applications. In this work, theoretical analysis is performed by finite element method (FEM), and the admittance characteristics of an A1 mode resonator and displacement of transverse modes near the resonant frequency (*f*_r_) are investigated. We propose a novel Dielectric-Embedded Piston Mode (DEPM) structure, achieved by partially etching a piezoelectric film filled with SiO_2_, which can almost suppress the transverse modes between the resonant frequency (*f*_r_) and anti-resonant frequency (*f*_a_) when applied on ZY-cut LiNbO_3_-based A1 mode resonators. This indicates that compared with Broadband Piston Mode (BPM), Filled-broadband Piston Mode (FPM) and standard structures, the DEPM structure is superior. Furthermore, the design parameters of the resonator are optimized by adjusting the width, depth and filled materials in the etched window of the DEPM structure to obtain a better suppression of transverse modes. The optimized A1 mode resonator using a DEPM structure exhibits a transverse-free response with a high *f*_r_ of 3.22 GHz and a large *K*^2^ of ~30%, which promotes the application of A1 mode devices for use in 5G RF front-ends.

## 1. Introduction

Acoustic resonators are one of the most indispensable components of radio-frequency (RF) front-ends used in mobile communications due to their low cost, small size and design flexibility [1,2,3,4,5]. With the arrival of Fifth Generation (5G) mobile communication, traditional acoustic resonators, mainly surface acoustic wave (SAW) and bulk acoustic wave (BAW) resonators, face higher technical challenges. The frequency of SAW resonators is limited by the accuracy of lithography, making it difficult to meet high-frequency application scenarios above 3 GHz [6,7,8,9,10,11]. In contrast, the frequency of BAW resonators is determined by the AlN piezoelectric film thickness, enabling higher frequencies to be achieved by controlling the film thickness, but it still cannot meet the demand for a broad bandwidth in 5G mobile communication because of the limited electromechanical coupling coefficient (*K*^2^) of AlN [12,13,14,15]. Alternatively, first-order antisymmetric (A1) lamb mode resonators possess the characteristics of both a high frequency and large *K*^2^, and to some extent, they are more adaptable than SAW and BAW resonators for applications in a sub-6 GHz wideband scenario [16,17,18,19,20,21]. Furthermore, it has gradually become a research hotspot in recent years. Nevertheless, one of the biggest challenges for A1 mode resonators is the fact that the presence of spurious modes in them acts as a critical bottleneck for their potential applications, since these spurious modes can lead to increased in-band ripples, higher losses, and a decline in overall performance. Therefore, how to weaken or eliminate spurious modes is a key issue in the development of high-performance A1 mode resonators [22,23,24,25].

To suppress spurious modes of A1 mode resonators, several methods have been studied. Plessky found that the A1 mode’s higher-order spurious mode (A1-3 mode) can be eliminated by adjusting metallization. However, at the same time, the magnitude of S0 and A0 spurious modes between the resonant frequency (*f*_r_) and anti-resonant frequency (*f*_a_) increased undesirably [26]. To address spurious modes in LiNbO_3_ A1 mode resonators, Songbin Gong demonstrated a method wherein the top electrodes are fully embedded in the piezoelectric film, and this design can effectively suppress the spurious modes caused by electrical and mechanical loadings [27]. Naumenko’s research showed that spurious modes of A1 mode resonators can be suppressed by adjusting the orientation and thickness of the piezoelectric film based on analysis of the BAW slowness surface [28]. In a nutshell, for their current study on spurious modes in A1 mode resonators, they essentially focused on high-order spurious modes or other lamb modes away from the resonance. However, little attention was paid to spurious modes that are known as transverse modes, which also require further study as they affect the performance of A1 devices [29,30,31].

This work proposes a dielectric-embedded piston mode (DEPM) structure, achieved by filling SiO_2_ in a partially etched ZY-cut LiNbO_3_ film, to suppress transverse modes in the A1 mode resonator. It is confirmed that the proposed structure can effectively suppress transverse modes of the A1 mode resonator based on theoretical and finite element method (FEM) analysis. The simulated admittance curve of the A1 mode resonator with the proposed structure shows a clean spectrum with a high *f*_r_ of 3.22 GHz and a large *K*^2^ of ~30%. Achievable performances of the proposed DEPM structure, previous structure and standard structure are compared, and the results indicate that the DEPM structure improves the suppression of transverse modes. In addition, the design parameters of DEPM structures are optimized by adjusting the width, depth and filled materials in etched windows to demonstrate the better suppression of transverse modes. This work also shows the simulated results of filters with the standard and DEPM structures, further proving the effectiveness of the proposed structure and providing a promising path for applications in 5G N77 bands for A1 mode filters.

## 2. Theoretical Analysis of Transverse Modes

Acoustic resonators can transform energy between mechanical and electrical domains by utilizing the piezoelectric effect. Wave motion and electrical behavior are coupled by constitutive equations, as follows [32]:

(1) $$T=c^{E}S-eE$$

(2) $$D=\varepsilon^{S}E+eS$$

where ***T*** and ***D*** are mechanical stress and electric displacement, respectively. They are expressed in terms of the mechanical strain ***S*** and the electric field ***E*** with the elastic stiffness constant *c^E^* at the constant electric field ***E***, dielectric constant *ε^S^* at the constant strain ***S*** and piezoelectric coefficient *e*. Note that the subscripts of these variables and material constants tensor are omitted for simplicity.

**Figure 1:**
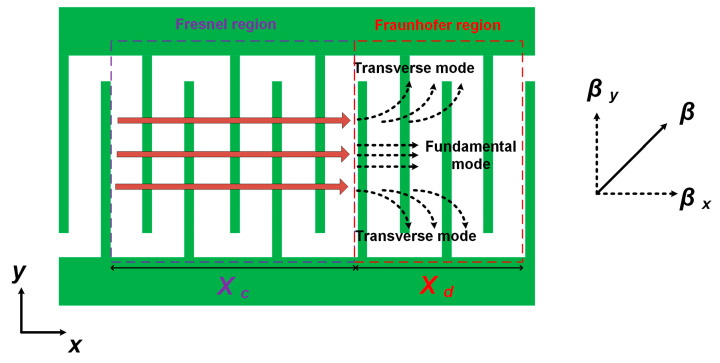
Propagation and diffraction of acoustic waves along with IDTs.

It is known that acoustic wave propagation can be determined through constitutive equations governed by the Maxwell equation and Newton equation of motion [33]. Diffraction is an inherent phenomenon of acoustic wave propagation. As shown in Figure 1, the acoustic wave propagates along with interdigital transducers (IDTs) in the *x*-direction, and diffraction occurs in the *y*-direction, which widens the propagation path of the acoustic wave and results in diffractive loss and spurious resonances. The region where acoustic waves propagate only in the *x*-direction is called the Fresnel region, and the region where acoustic waves propagate in both *x*- and *y*-directions with diffraction is called the Fraunhofer region. The length of the Fresnel region *X*_c_ is given by [33]:

(3) $$X_{c}=\frac{\left(1+\gamma \right)AP^{2}}{\lambda }$$

where *AP* and *λ* are the aperture and the wavelength of IDTs, respectively, and *γ* is a factor determined by the anisotropy of the piezoelectric film. For a specific piezoelectric film, the factor *γ* remains constant. Obviously, a larger *AP* and smaller *λ* with a larger derived *X*_c_ are recommended for IDTs’ design and fabrication, so that the length of the Fresnel region is extended to the utmost in order to avoid wave diffraction.

In practical devices’ fabrication, however, the infinite length of aperture leads to a broad Fraunhofer region on IDTs, which makes the occurrence of wave diffraction in acoustic resonators inevitable. The wave number in the *y*-direction (*β_y_*) grows with a decrease of *AP* in the Fraunhofer region, as shown in Figure 1. Additionally, transverse resonant modes will be generated between both sides of the busbars. Here, the wave number *β_y_* is given by:

(4) $$\beta_{y}=2j\times \frac{\pi }{AP}\;\left(j=0,\;1,\;2,\;3\ldots \right)$$

where *j* is the order of transverse modes.

**Figure 2:**
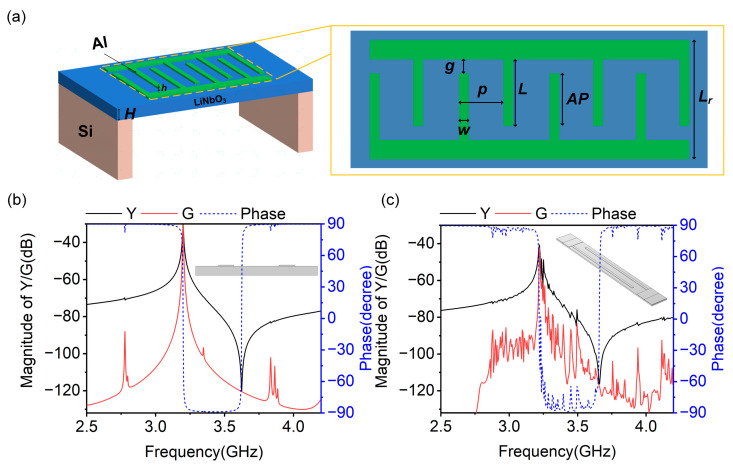
(**a**) Typical structure of the A1 mode resonator. (**b**) The 2D model in simulation and 2D FEM simulation admittance (Y)/conductance (G)/phase curves of the A1 mode resonator with typical structure. (**c**) The 3D model in simulation and 3D FEM simulation admittance (Y)/conductance (G)/phase curves of the A1 mode resonator with typical structure.

As shown in Figure 2a, the typical A1 lamb mode resonator consists of a suspended ZY-cut lithium niobate (LiNbO_3_, LN) film and aluminum (Al) IDTs that generate a lateral electric field to excite a strong A1 mode due to the high piezoelectric component *e*_24_. To guarantee the generation of the A1 mode, the electrode pitch *p* is significantly larger than the LN thickness *H*, and usually *H*/*p *< 0.2.

The resonance frequency depends on the geometry of the resonator (*p* and *L*_r_) and the acoustic velocity (*v*) of the expected mode, expressed as [34]:

(5) $$f_{ij}=\frac{v}{2}\sqrt{{\left(\frac{i}{p}\right)}^{2}-{\left(\frac{j}{L_{r}}\right)}^{2}}$$

where *f_ij_* is the resonance frequency of the *i*th-order longitudinal (*x*-direction) and *j*th-order transverse (*y*-direction) modes. For the A1 mode, it only propagates in the *x*-direction, and *i*, *j* are equal to 1 and 0, respectively. Thus, the resonance frequency for the A1 mode can be approximated as:

(6) $$f=\frac{v}{2p}$$

As an effective and universal method, FEM has been widely used for the simulation of acoustic resonators, and there have been extensive reports of its accuracy after decades of development. Furthermore, FEM modeling based on COMSOL Multiphysics provides a convenient and flexible way to build arbitrary structures. Therefore, FEM simulations of the A1 mode are carried out on the ZY-cut LiNbO_3_ to capture the acoustic characteristics by using the solid mechanics and electrostatics module in the COMSOL Multiphysics 5.6 software [35,36]. Considering the IDT periodicity of the A1 resonator, the FEM models in this work are established by using the periodic boundary conditions U_L_ = U_R_ along the *x* axis to reduce time consumption and memory consumption, where U_L_ and U_R_ respectively represent field variables on the left and right surfaces [37,38]. The key parameters of the resonator are explained in Table 1. In the following article, *K*^2^ is derived from *K*^2^ = (π^2^/4) × (*f*_a_ − *f*_r_)/*f*_a_. In the 2D simulation, due to the fact that the 2D model is established based on the *x* direction and *z* direction, the diffraction of acoustic waves cannot be reflected in the simulation (there is no *β_y_*). The A1 mode at 3.2 GHz has a large *K*^2^ of ~28.9%, achieving a spurious-free response, as shown in Figure 2b. In the 3D simulation, the effects and variations along all three directions (*x*, *y*, and *z*) including *β_y_* are captured, and the perfect matching layers (PMLs) are given to the left and right ends with a busbar width to reduce the size of the model and to suppress the boundary reflections. The A1 mode at 3.22 GHz has a large *K*^2^ of ~30%. Different from the spurious modes near resonance, e.g., S0-n and A0-n modes, several transverse modes appear between *f*_r_ and *f*_a_, as shown in Figure 2c. The features of the A1 mode can be basically matched in both 2D and 3D simulations, including *f*_r_ and *K*^2^, differing only in the presence or absence of transverse modes. These obvious transverse modes are successfully calculated in the 3D simulation, showing an amplitude of more than 3 dB, which is not acceptable for the development and application of high-performance A1 mode resonators.

**Figure 3:**
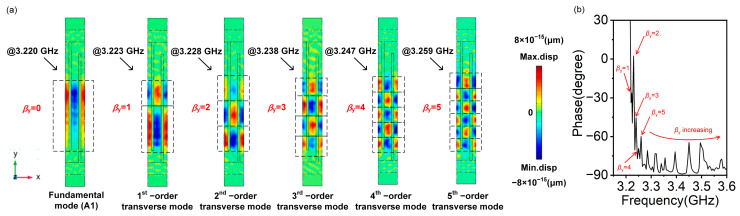
(**a**) The displacement in the *x*-direction of A1 mode and 1st~5th-order transverse modes at different frequency positions. (**b**) Different locations of 1st~5th-order transverse modes in the spectrum (amplified phase curves in Figure 2c).

To further analyze the displacement of the calculated transverse modes near *f*_r_, we determine the high-order transverse modes present in the A1 mode resonator using 3D FEM analysis. The simulation parameters remain the same as in Table 1. Figure 3a shows the displacement in the *x*-direction of the A1 mode and 1st~5th-order transverse modes at different frequency positions, and Figure 3b illustrates the different locations of these transverse modes in the spectrum. Note that higher-order transverse modes (6th, 7th, 8th…) are also present in the admittance curve and that their displacements are not shown here. When there is no transverse wave number (*β_y_* = 0), the resonant mode is the fundamental (A1) mode. Transverse resonant modes start to appear when *β_y_* ≥ 1. As *β_y_* increases, the order of transverse modes increases, and it can be seen that the diffraction of acoustic waves gradually becomes apparent.

## 3. Results

### 3.1. The Suppression Results for the Previous and Derived Structures

**Figure 4:**
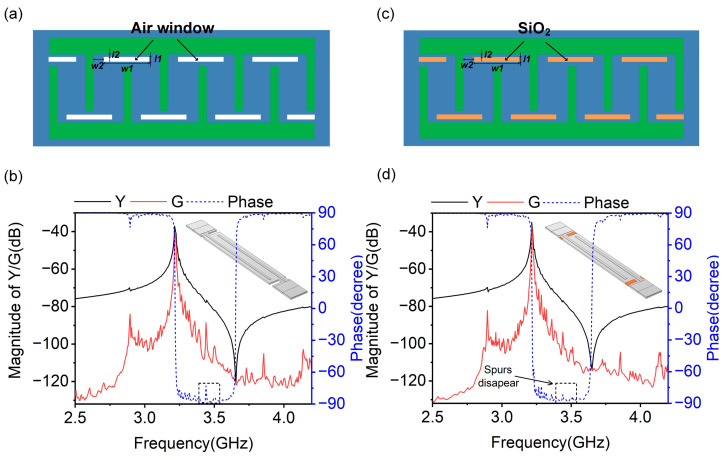
(**a**,**c**) Conceptual diagrams of the A1 mode resonator with BPM and FPM structures. (**b**,**d**) 3D models in simulations and 3D-simulated admittance/conductance/phase curves of the A1 mode resonator with BPM and FPM structures; the structure has *w*1 = 6 μm, *w*2 = 1 μm, *l*1 = 2 μm, and *l*2 = 1 μm.

The transverse mode can be suppressed by adding a functional area between the active region and busbars, working similarly as a free boundary condition, which weakens the acoustic wave reflection in the aperture direction [39]. In the previous work, researchers used the method of placing air windows between the active region and busbars to suppress the transverse modes of the A1 mode resonator, called the Broadband Piston Mode (BPM) structure, as shown in Figure 4a [40]. Figure 4b shows the 3D FEM model and 3D-simulated admittance/conductance/phase curves of the A1 mode resonator with a BPM structure. In order to observe the basic features of the resonator expediently, including the *f*_r_, *f*_a_, admittance ratio and transverse modes, both the admittance and conductance were shown in this work in the format of their magnitude dB (Y/G (dB) = 20 × log10|Y/G|). As can be clearly seen in the conductance or phase curves, the BPM structure indeed improves the suppression of transverse modes compared to the results of the standard structure (Figure 2c). However, there are still several spurs in the spectrum caused by transverse modes, which need to be further suppressed. To this end, we propose another structure called Filled-broadband Piston Mode (FPM), according to BPM. Figure 4c shows a diagram of the proposed structure, in which SiO_2_ with a thickness of *H* is filled in air windows. Figure 4d shows the 3D FEM model and 3D-simulated admittance/conductance curves of the A1 mode resonator with the FPM structure, where *w*1, *w*2, *l*1 and *l*2 are set to be the same as for the BPM structure. The simulation results show that the FPM structure has a slightly improved suppression of transverse modes when compared with the BPM structure, since less spurs are caused by transverse modes occurring in the admittance curve between *f*_r_ and *f*_a_. However, both BPM and FPM structures are insufficient for realizing transverse-free A1 mode resonators.

### 3.2. The Suppression Results for the Proposed Strutcure

**Figure 5:**
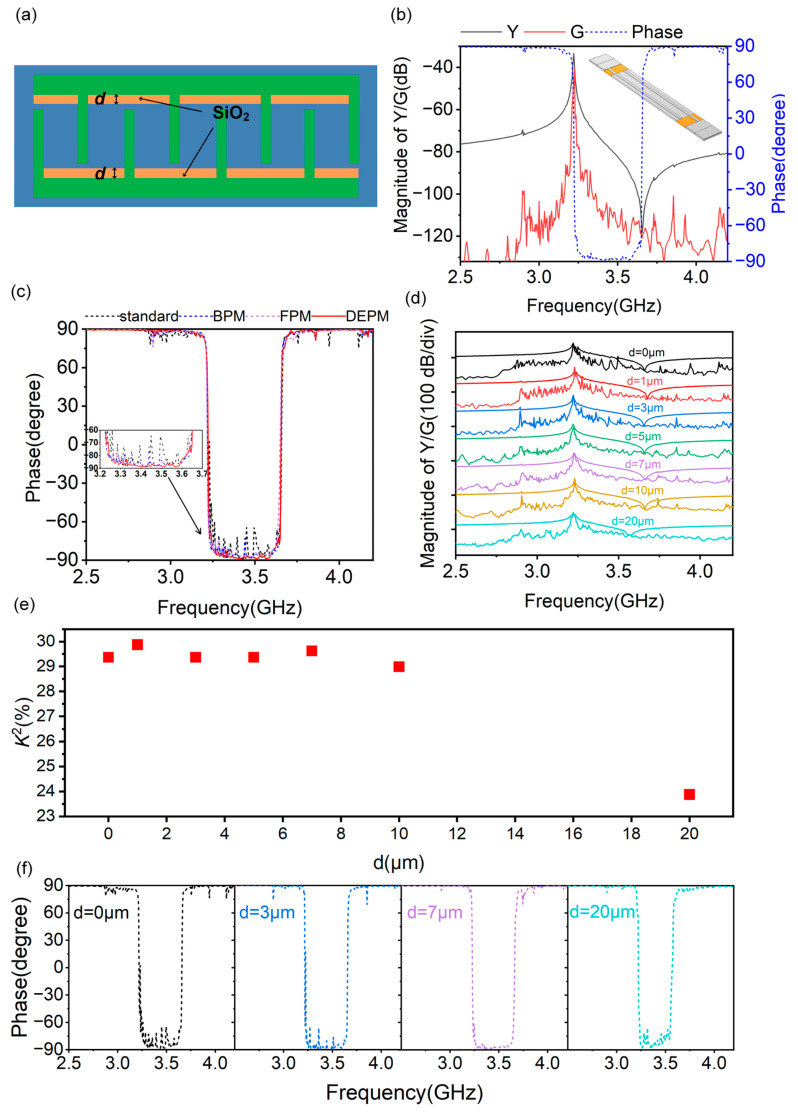
(**a**) Conceptual diagram of the A1 mode resonator with DEPM structure. (**b**) Simulated admittance/conductance curves of the A1 mode resonator with *d* = 5 μm. (**c**) Simulated phase curves of the A1 mode resonator with the DEPM, BPM, FPM and standard structures. (**d**) Simulated admittance/conductance curves of the A1 mode resonator with different *d*. (**e**) Simulated *K*^2^ of the A1 mode resonator with different *d*. (**f**) Simulated phase curves of the A1 mode resonator with *d* = 0 μm, 3 μm, 7 μm and 20 μm.

Therefore, we propose a novel structure to further suppress transverse modes, which is called the Dielectric-Embedded Piston Mode (DEPM) structure, as shown in Figure 5a. In this structure, all or part of the piezoelectric films between the active region and busbars are etched and then filled with SiO_2_, which can even extend to the active region. The width of SiO_2_ is *d*, and other parameters in the resonator remain consistent with the standard structure (Figure 2a). Figure 5b depicts the 3D model in simulations and 3D-simulated admittance/conductance/phase curves of the A1 mode resonator with *d* = 5 μm. One can see that the A1 mode at 3.22 GHz has a large *K*^2^ of ~30%, and such a high *K*^2^ is nearly comparable to those reported in previous studies by others, while most of the transverse modes have been suppressed. The result demonstrates that the DEPM structure is better at transverse modes’ suppression than the BPM, FPM and standard structures are. To quantify the suppression, the phase curves of A1 mode resonators are shown as a metric. The generation of an acoustic mode inevitably leads to a change in phase, and the strength of the acoustic mode correlates with the amplitude of the phase change. As shown in Figure 2 and Figure 5, the phase of the transverse modes reaches ~−60° for the standard structure, and the phase of the transverse modes reaches ~−70° for the BPM and FPM structures, while for the DEPM structure, the phase of the transverse modes is ~−90°. The phase curve of the A1 mode resonator with the DEPM structure has less phase variation compared to that with the BPM, FPM and standard structures, which further demonstrates the suppression of transverse modes. On the other hand, the use of SiO_2_ can reduce the Temperature Coefficient of Frequency (TCF) of the resonator, facilitate the frequency stability of the A1 mode resonator and broaden its applicability [41,42].

In the following discussion, the influence of *d* on acoustic wave propagation is analyzed. The admittance/conductance curves under *d* from 0 to 20 μm are illustrated in Figure 5c. The amplitude of the transverse modes is appropriately reduced with the increase of *d*. However, it is inappropriate for an excessively large *d*, as it can lead to a decrease in *K^2^*. As shown in the figure, when *d* ranges from 5 to 10 μm, transverse modes have a better suppression effect; meanwhile, the A1 mode maintains a large *K*^2^, only showing neglectfully tiny, spurious modes in the broadband frequency response. This behavior is even more significant when observing the phase curve of A1 mode resonators according to phase variation, as shown in Figure 5d.

**Figure 6:**
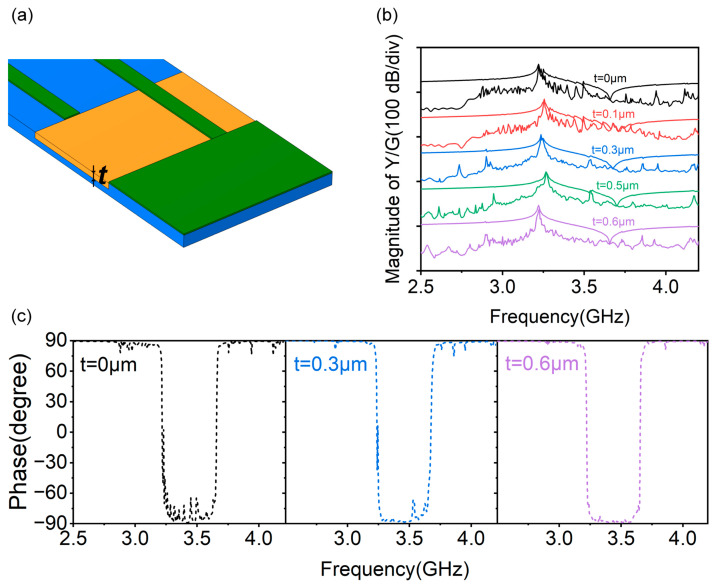
(**a**) Conceptual diagram of DEPM structure with depth *t* of SiO_2_. (**b**) 3D-simulated admittance/conductance curves of the A1 mode resonator with different *t*. (**c**) 3D-simulated phase curves of the A1 mode resonator with *t* = 0 μm, *t* = 0.3 μm and *t* = 0.6 μm.

The depth *t* of the SiO_2_ is also taken into consideration to optimize the transverse mode suppression of the DEPM structure. A conceptual diagram of the A1 mode resonator with *t* is drawn in Figure 6a, where *d* = 5 μm and the other parameters in the resonator remain consistent with the standard structure (Figure 2a). Figure 6b illustrates the 3D-simulated admittance/conductance curves of the A1 mode resonator as a function of *t* from 0 to 0.6 μm. By comparing the results, the transverse modes gradually weaken as *t* increases, and this means that it is better to fill SiO_2_ into the completely penetrated ZY-cut LiNbO_3_ film in order to suppress transverse modes. This suppression effect is also found in phase curves. As shown in Figure 6c, the phase variation caused by transverse modes decreases as t increases, until the phase curves become stable.

**Figure 7:**
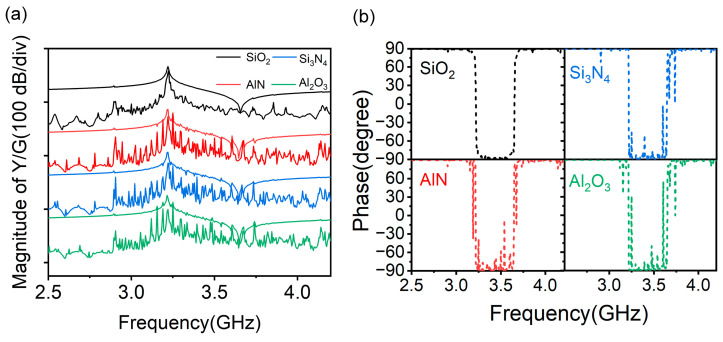
(**a**) Simulated admittance/conductance curves of the A1 mode resonator with different filled dielectric materials. (**b**) Simulated phase curves of the A1 mode resonator with different filled dielectric materials.

To verify the suitability of other dielectric materials for the DEPM structure, the use of other dielectric materials (Si_3_N_4_, AlN and Al_2_O_3_) is also investigated. Figure 7a,b shows, respectively, the 3D-simulated admittance/conductance and phase curves of the A1 mode resonator with SiO_2_, Si_3_N_4_, AlN and Al_2_O_3_ in the DEPM structure. The results suggest that the *f*_r_, *f*_a_, *K*^2^ and admittance ratio stay constant from the calculated admittance, while SiO_2_ shows a significantly better transverse modes suppression when compared with other dielectric materials, which can be particularly seen in the phase curves.

### 3.3. The Suppression Results in Applicable Filters

**Figure 8:**
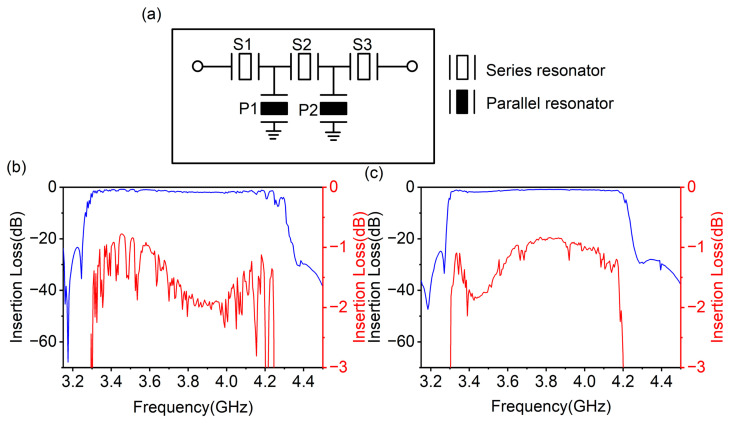
(**a**) Topology of simulated filters. (**b**) The insertion-loss curve of the simulated adder-type filter with the standard structure. (**c**) The insertion-loss curve of the simulated adder-type filter with the DEPM structure.

Moreover, we provide the simulation results of conventional adder-type filters with the proposed DEPM structure and standard structure in a 5G N77 band (3.3 GHz~4.2 GHz). Figure 8a illustrates the topology of the simulated filters, consisting of three series resonators (abbreviated as S1–S3) and two parallel resonators (abbreviated as P1, P2).

All simulations of filters are accomplished through the electrical characteristics of conductive paths and package parasitics using a standard surface-mounted device (SMD), which connects with the acoustic characteristics of series and parallel resonators. Figure 8b,c show the frequency response curves of the filters with the standard and DEPM structures. The features of both filters suggest a passband of 3.3 GHz~4.2 GHz, a minimum insertion loss of 0.8 dB and an out-of-band rejection of over 20 dB. However, when comparing the two figures, the filter with the standard structure has a lot of spurs in the passband, while one can clearly see that those are weakened in the filter with the DEPM structure. This proposed structure applied in filters has successfully suppressed the transverse modes of resonators and significantly improved the flatness of the passband, confirming that high-performance filters are suitable for a 5G N77 band.

## 4. Conclusions

In this work, the suppression of the transverse modes of LiNbO_3_ A1 mode resonators is discussed. We propose a Dielectric-Embedded Piston Mode (DEPM) structure in A1 mode resonators, and the simulated admittance curve of the A1 mode resonator with the proposed structure shows a clean spectrum with a high frequency *f*_r_ of 3.22 GHz and a large *K*^2^ of ~30% obtained by FEM analysis. In addition, the appropriate width and depth of SiO_2_ in the structure are studied and recommended in order to suppress the transverse modes. Compared with the previous Broadband Piston Mode (BPM), proposed Filled-broadband Piston Mode (FPM) and standard structures, this indicates that the DEPM structure is superior to the BPM, FPM and standard structures. Simultaneously, a simulated adder-type filter with the DEPM structure in a 5G N77 band was presented and proven to improve the flatness of the passband in the filter. This technology is expected to solve conundrums related to transverse modes and provide a promising path for future applications in 5G new radios.

## Figures and Tables

**Table 1:** The key parameters of a typical A1 mode resonator.

Sym.	Parameter	Value	Sym.	Parameter	Value
*p*	Pitch of electrodes (μm)	4	*AP*	Aperture (μm)	40
*w*	Width of electrodes (μm)	1	*L_r_*	Length of resonators (μm)	60
*g*	Gap length (μm)	5	*H*	LiNbO_3_ thickness (nm)	600
*L*	Length of electrodes (μm)	45	*h*	Al thickness (nm)	75

## Data Availability

The data presented in this study are available on request from the corresponding author.
